# Blood swabs represent an alternative sample matrix for detection of antibodies against classical swine fever virus during surveillance in wild boar

**DOI:** 10.1007/s11259-026-11292-3

**Published:** 2026-05-25

**Authors:** Denise Meyer, Sandra Blome, Lia Ebner, Paul Becher

**Affiliations:** 1 EU and WOAH Reference Laboratory for Classical Swine Fever, Institute of Virology, University of Veterinary Medicine Hannover, Buenteweg 17, Hannover, 30559 Germany; 2 Institute of Diagnostic Virology, Federal Research Institute for Animal Health, Suedufer 10, Greifswald, 17493 Germany

**Keywords:** Classical swine fever, African swine fever, Wild boar, Blood swabs, Serology, Antibody detection

## Abstract

Passive surveillance of fallen wild boar represents an important tool for detecting notifiable diseases such as Classical swine fever (CSF) and African swine fever (ASF) which are showing comparable pathological findings. For differential diagnosis, it is advantageous to use one sample matrix for testing of both diseases. Especially, if no serum sample can be collected from wild suids, blood swabs represent an alternative matrix in ASF diagnosis. On this basis, the present study aimed to analyze blood swabs serologically using CSF antibody ELISAs and virus neutralization tests. In total, twenty-nine CSF antibody positive samples and one-hundred CSF antibody negative samples were examined. The results show that this sample matrix is also suitable for detecting antibodies against CSF virus (CSFV). In general, the sensitivity of blood swabs is slightly reduced compared to the analysis of serum, which can affect the classification of samples taken during early seroconversion. However, samples collected at  ≥ 21 days post CSFV infection are tested safely antibody positive.

**Supplementary Information:**

The online version contains supplementary material available at 10.1007/s11259-026-11292-3.

## Introduction

Classical swine fever (CSF) is a severe systemic disease that affects both domestic pigs and wild suids (Blome et al. 2017; Postel et al. 2018; Ganges et al. 2020). As clinical and pathological findings of CSF are often comparable to those of African swine fever (ASF), differential diagnosis is performed. Outbreaks of both diseases are associated with high economic losses in the pig industry due to the high lethality of the diseases and a widely conducted stamping out policy along with transport and trade restrictions (Meuwissen et al. 1999; Saatkamp et al. 2000; Moennig and Becher 2015). While in the European Union (EU) the last sporadic outbreaks of CSF occurred in Latvia in 2015, ASF has emerged in several European countries over the last decade. However, it is not unlikely that CSF will be reintroduced in CSF free countries as global exchange and trade are steadily increasing. Passive surveillance of fallen wild boar plays an important role in early detection of disease introduction. In this context, cooperation with hunters in sampling of fallen wild boar or carcasses is essential (Carlson et al. 2018). For both diseases, whole blood and organ samples are recommended for the detection of viral genomes. In addition, whole blood is used for the preparation of serum, which is tested for antibodies against CSF virus (CSFV) and ASF virus (ASFV). However, in most cases, it is not possible to obtain blood samples of good quality and in sufficient quantity from fallen wild boar. To overcome this issue, pragmatic sampling strategies have been tested for ASF including FTA cards, filter papers and quick-drying swabs (Blome et al. 2014; Petrov et al. 2014; Braae et al. 2015; Randriamparany et al. 2016; Carlson et al. 2018). For diagnosis of CSF, quick-drying blood swabs were analyzed exclusively for the presence of viral genome, with the results being comparable to those of the original blood samples (Petrov et al. 2014). So far, the application of blood swabs to detect antibodies has only been described for ASF (Blome et al. 2014) but not for CSF. Therefore, the present study aimed to evaluate the application of blood swabs for the detection of antibodies against CSFV by ELISA and virus neutralization. Since the number of available samples was relatively small, a proof-of-concept study without complete diagnostic validation was conducted.

## Materials and methods

### Serum and blood swabs

Serum and EDTA blood samples, which are positive for antibodies against CSFV, were obtained from the sample collection of the EU and WOAH Reference Laboratory for CSF (Institute of Virology, University of Veterinary Medicine Hannover, Germany) and the National Reference Laboratory for CSF (Institute of Diagnostic Virology, Friedrich-Loeffler-Institute, Federal Research Institute for Animal Health, Greifswald, Germany). The samples were collected from pigs infected with CSFV strains of different genotypes. A total of twenty-nine serum and blood samples were analyzed, including serial-derived samples collected from four domestic pigs (Online Resource 1). Moreover, CSF antibody negative blood samples taken from a wild boar, warthog and red river hog (kept under experimental conditions) were spiked with a serum that was collected at 183 days post infection (dpi) using the CSFV isolate Diepholz (genotype 2.3). This serum contains a high antibody titer against a genotype 2 strain, which has been most frequently detected during recent field outbreaks. For this purpose, serum was added to the blood samples to reach a dilution of 1:4, 1:8, 1:16 and 1:32. In addition, fifty CSF antibody negative samples each from domestic pigs and wild boar were examined.

For the preparation of dried blood- or serum swabs, GenoTube Livestock swabs (Thermo Fisher Scientific, Lelystad, The Netherlands) were completely soaked for 1 min with blood or serum. To simulate the transport of field samples, the swabs were left overnight at room temperature (21–23 °C). Subsequently, 0.25 cm of the swab was cut off and added directly to 270 µl sample dilution buffer of the corresponding ELISA kit (100 µl per reaction; double approach for each ELISA). For analysis by VNT, the swab sample was added to 130 µl cell culture medium since the starting sample amount per double approach is 40 µl. After incubation in an overhead swivel for 1 h at room temperature the collected supernatant was tested in ELISA or VNT. The duration between completion of swab preparation and testing in the serological assay was 24 h. Serum swabs were included in the analysis to estimate the dilution effect resulting from incubation of the swabs in dilution buffer or cell culture medium. For each serological assay, all samples were analyzed in duplicates.

### Detection of antibodies against CSFV by ELISA and virus neutralization test

For the detection of antibodies against CSFV, the samples were tested in two commercial CSF antibody ELISAs (IDEXX CSFV Ab, IDEXX Switzerland GmbH, Liebefeld-Bern, Switzerland; ID Screen^®^ Classical Swine Fever E2 Competition, Innovative Diagnostics, Grabels, France) according to the manufacturer’s recommendations, which are widely used by national reference laboratories. Both assays are not validated for the sample matrix blood. In this study, 100 µl supernatant of each swab samples per cavity was used. The original serum samples were diluted as described in the protocol of the manufactures.

The VNT was performed according to the protocol described in the Manual of Diagnostic Tests for Detection of CSF, which is available on the website of the EU Reference Laboratory for CSF (https://www.tiho-hannover.de/en/clinics-institutes/institutes/institute-of-virology/eu-and-woah-reference-laboratory/diagnostic-methods), using the CSFV reference strains Alfort/187 and Diepholz and the porcine kidney cell line PK 15 (CCLV0051), which was obtained from the Collection of Cell Lines in Veterinary Medicine (CCLV, Friedrich-Loeffler-Institute, Federal Research Institute for Animal Health, Greifswald, Germany).

## Results

Serum represents the preferred sample matrix for detection of antibodies against CSFV. To test whether blood swabs are a suitable alternative, swabs were soaked with blood taken from pigs after experimental infection with CSFV and were initially analyzed only in the IDEXX CSFV Ab ELISA. The samples were collected at ≥ 21 dpi except for one sample, which was taken 17 dpi. In addition to the blood swab samples, the corresponding serum samples and a swab soaked with this serum were included in the analysis as controls. The comparison between serum and the serum swab shows an average decrease of approximately 5% blocking percentage in the CSFV Ab ELISA (influence of the swab). When analyzing blood swabs, the blocking percentage is significantly lower (approximately 9%) compared to the results detected for the corresponding serum samples (influence of the sample matrix blood; Table 1).

**Table 1:** Comparison of different sample matrices tested in the CSFV Ab ELISA (IDEXX)

Sample matrix	Number of samples tested positive	Mean value [blocking %] (*n* = 15)	Minimum value [blocking %] (*n* = 15)	Maximum value [blocking %] (*n* = 15)	Standard deviation	*p*-value in comparison to serum*
Serum	15	87.05	67.89	95.03	6.99	-
Serum swab	15	82.32	66.07	92.45	7.44	0.1050
Blood swab	15	79.25	61.15	92.05	8.35	0.0150

cut-off value for a CSF antibody positive sample: ≥ 40% blocking; * = statistical analysis using the unpaired t-test. Definition for statistical significance = *p*-values < 0.05: significant. The statistical analysis was performed using GraphPad Prism 10.4.1

**Fig. 1:**
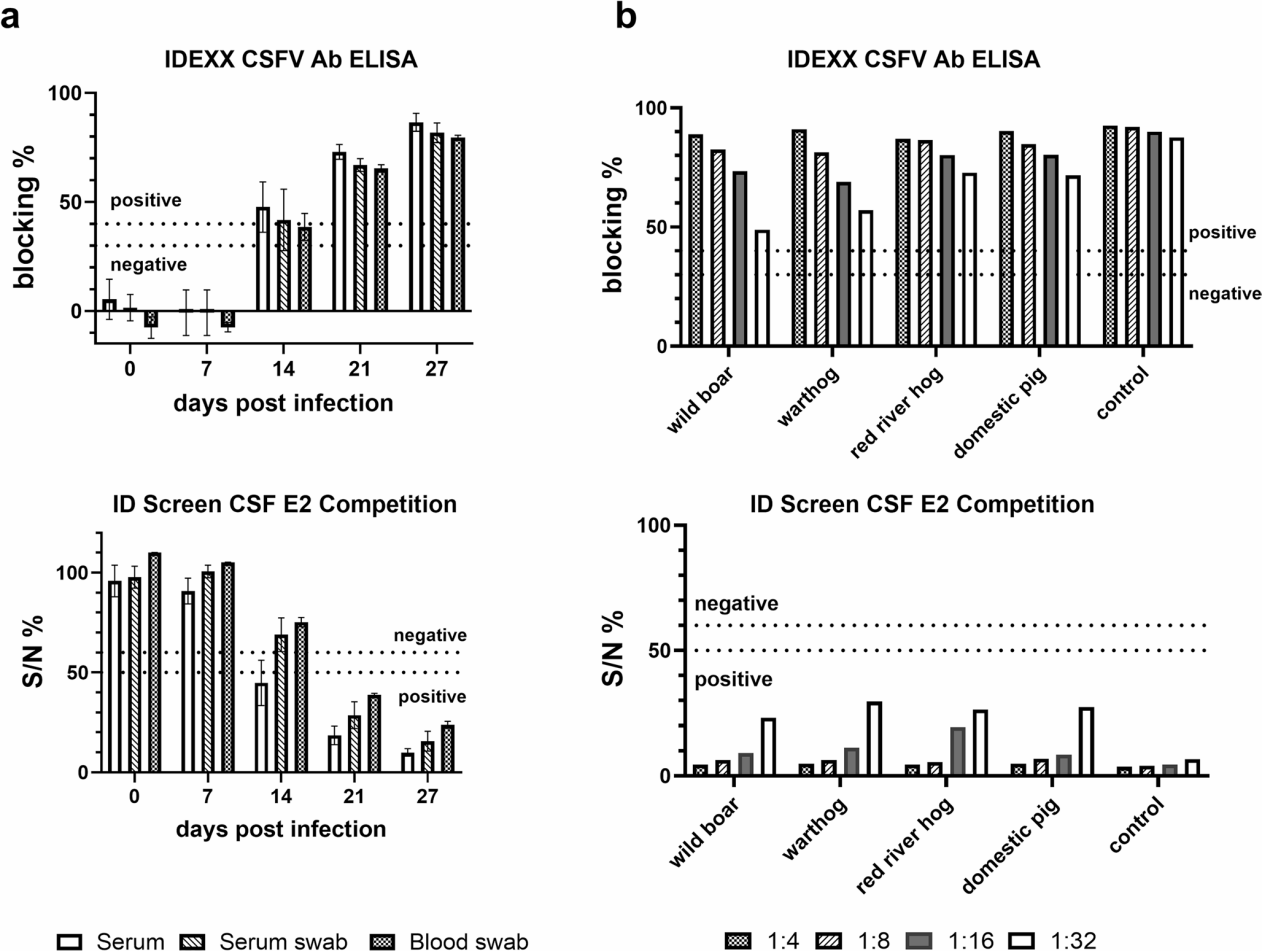
Detection of antibodies against CSFV using two commercial CSF antibody ELISAs. (**a**) Blood swabs were analyzed in comparison to serum swabs and serum. The samples were collected from four domestic pigs at different days post infection (dpi), including 0 dpi (*n* = 4), 7 dpi (*n* = 4), 14 dpi (*n* = 3), 21 dpi (*n* = 4), 27 dpi (*n* = 4). The mean values obtained for the samples from four animals and the standard deviations are shown for each time point (0, 7, 21 and 27 dpi) and sample matrix. Samples collected at 14 dpi were available from only three out of four animals (*n* = 3). (**b**) Analysis of blood samples from wild suids. Blood collected from wild suids (one wild boar, one warthog and one red river hog) and from one domestic pig were spiked with serum that contains antibodies against CSFV strain Diepholz and were analyzed by the two CSF antibody ELISAs. Four different serum dilutions (1:4, 1:8, 1:16 and 1:32) were applied. As a control, CSF antibody positive serum (against the CSFV strain Diepholz) was diluted using the same dilution factors in CSF antibody negative serum (control). The dotted lines in the diagrams depict the cut-off values according to the manufactures descriptions (IDEXX CSFV Ab ELISA: blocking % ≥ 40% = positive for antibodies against CSFV, blocking % ≤ 30% = negative for antibodies against CSFV, 30% < blocking % < 40% = doubtful for antibodies against CSFV; ID Screen CSF E2 Competition: S/N % ≤ 50% = positive for antibodies against CSFV, S/N % > 60% = negative for antibodies against CSFV, 50% < S/N % ≤ 60% = doubtful for antibodies against CSFV)

However, all analyzed blood swabs, which were collected at ≥ 21 dpi, as well as the sample collected at 17 dpi, were safely detected positive for antibodies against CSFV. The significant loss of sensitivity might play a role in sample classification if the samples were taken at an early stage after infection, which is characterized by a low antibody level against the infectious agent. Therefore, samples were analyzed that had been taken from four animals on different days after infection (0, 7, 14, 21 and 27 or 28 dpi). The detection of antibodies against CSFV in the serum samples starts in both CSF antibody ELISAs at 14 dpi. Here, sample material from three out of four animals was available. According to the detected difference observed between serum and blood swabs (Table 1), one of these three blood swabs, taken at 14 dpi, was tested negative for antibodies against CSFV in the IDEXX CSFV Ab ELISA (Fig. 1a; Online Resource 2). In comparison to this, all these samples were tested negative in the second CSF antibody ELISA (ID Screen CSF E2 Competition). However, samples that were taken from reconvalescent animals (≥ 21 dpi) were safely detected positive for antibodies against CSFV using both commercial ELISA kits.

**Fig. 2:**
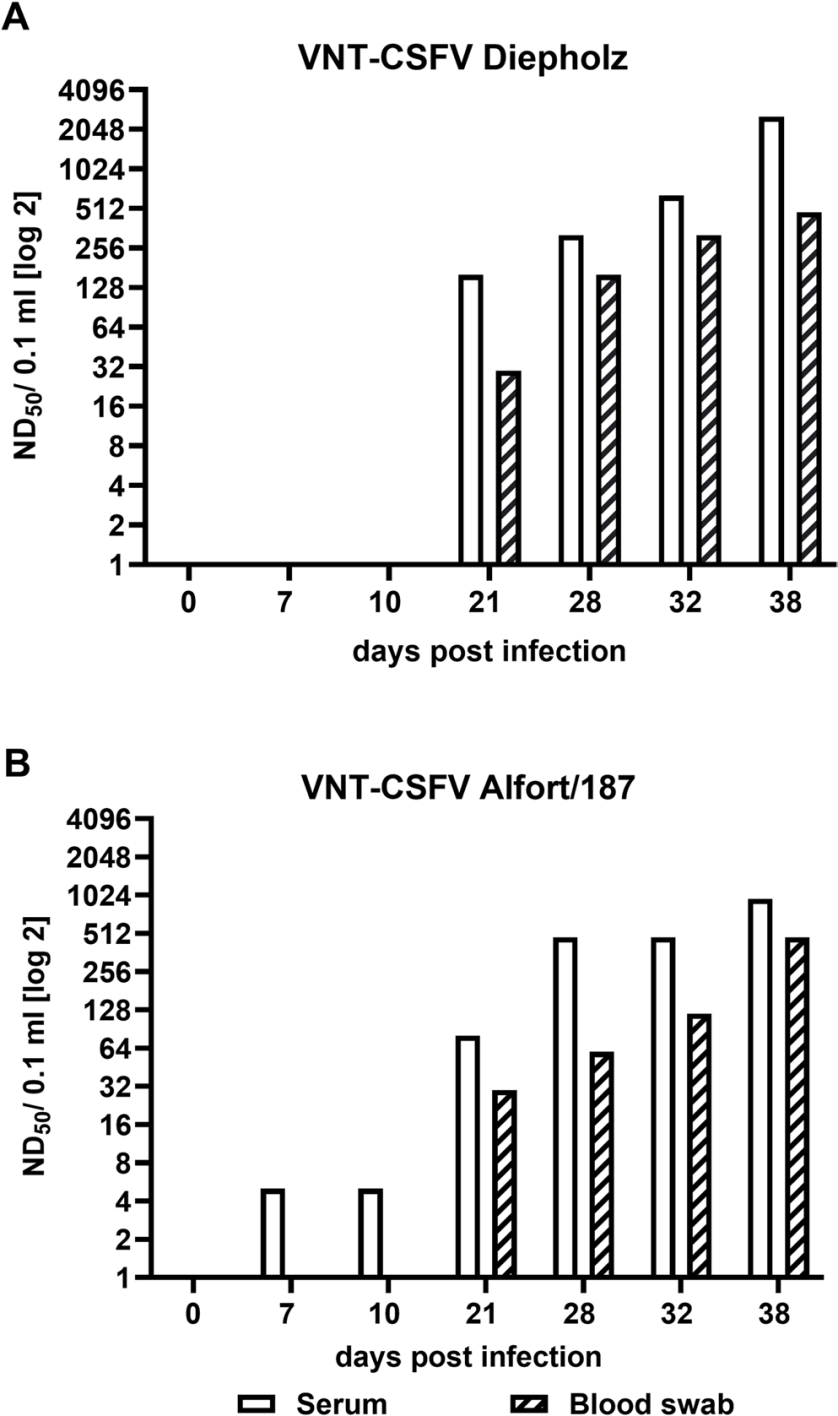
Comparative analysis of serum and blood swab in the CSF virus neutralization test (VNT) using the CSFV reference strains Diepholz (genotype 2.3) and Alfort/187 (genotype 1.1). Samples were collected at different time points after experimental infection of a domestic pig with CSFV (Isolate CSF1059). Blood swabs were incubated in 130 µl cell culture medium with antibiotics before use. Titers of neutralizing antibodies were calculated in ND_50_/100 µl

Since CSF has not been detected within the last decade in the EU, no field samples collected from wild suids are currently available. Therefore, blood samples from wild suids were spiked with serum that contains antibodies against CSFV. All blood swabs were detected positive for antibodies against CSFV in both ELISAs. The respective dilution effect of the serum could be demonstrated comparatively in all tested blood swabs, which confirms that no inhibitory side effects are detected when analyzing blood swabs from wild suids (Fig. 1b). According to the Manual of Diagnostic Tests for Detection of CSF of the EU Reference Laboratory for CSF (https://www.tiho-hannover.de/en/clinics-institutes/institutes/institute-of-virology/eu-and-woah-reference-laboratory/diagnostic-methods), doubtful or positive results detected in a CSF antibody ELISA should be confirmed by VNT. The application of blood samples in the VNT was tested as an example by using serum and blood swabs collected from one domestic pig at different time points after experimental infection (isolate CSF1059, animal ID 429; Online Resource 1). Neutralizing antibody titers were determined in two CSFV-specific VNTs using the CSFV reference strains Diepholz (genotype 2.3) and Alfort/187 (genotype 1.1). Compared to the serum samples, the titers of neutralizing antibodies detected in the blood swabs are lower (up to eightfold). Antibody titers in the VNT using a CSFV strain of genotype 2 (CSFV Diepholz) are higher than the antibody titers against genotype 1 CSFV strain Alfort/187, as the inoculum used for the experimental infection is a genotype 2 virus isolate. No cytotoxic effects on the cells were detected in the VNT. As shown for the ELISA results, samples collected at ≥ 21 dpi are safely detected positive for neutralizing antibodies (Fig. 2).

## Discussion

As several European countries are currently affected by ASF, both active and passive surveillance in wild boar is being carried out. In this context, the analysis of samples collected from wild boar includes not only ASF diagnosis but also the differential diagnosis from other porcine diseases such as CSF. During the last decade, CSF has not been detected in the EU, but extensive global exchange and international trade pose a serious and continuous risk for reintroduction of the disease, as it occurred in Japan in 2018 after a 26 years period of absence of CSF (Postel et al. 2019). The disease is still circulating in Japan as well as in other countries in Asia, and South and Central America (Ganges et al. 2020). Therefore, passive surveillance of wild boar plays a key role in early detection of CSF introduction. Previous studies on ASF showed that dried blood swabs represent a valuable alternative for sampling of fallen wild boar when the preferred sample material cannot be obtained. As this sampling method showed promising results in detecting CSFV and ASFV genomes (Petrov et al. 2014) and in detecting antibodies against ASFV (Blome et al. 2014), it was interesting to evaluate the application of blood swabs in CSF specific serological assays. The number of samples examined in this proof-of-concept study is rather low because no field samples collected from wild boar infected with CSFV are currently available as the EU Member States are CSF-free. Therefore, a large-scale field validation based on the present proof-of-concept study is still required. In addition, the experimentally antibody spiked blood swabs of wild suids do not replace the analysis of samples collected from naturally infected wild boars. Nevertheless, the present proof-of-concept study showed that antibodies against CSFV can be reliably detected from blood swabs taken  at ≥ 21 dpi. This good correlation was also detected in previous studies on ASF (Carlson et al. 2018). However, direct comparison of serum and blood swabs showed that blood samples with low CSF antibody concentrations might not be reliably identified as antibody positive. The onset of the humoral immune response against CSFV starts at 10–14 dpi (Blome et al. 2017; Ganges et al. 2020). At this stage the animals are often CSFV genome positive. Although the use of blood swabs has its limitation regarding early disease recognition based on antibody detection, this sample matrix can certainly be used for the detection of viral genome (Petrov et al. 2014). Thus, blood swabs should be used when the generally preferred sample matrix serum is not available. It represents a pragmatic sampling method, especially for hunters who can be involved in active surveillance, which can result in increased number of samples tested. Taken together, the results of the present study show that dried blood swabs can be used as a single sample matrix for the differential diagnosis of both diseases, CSF and ASF with minor limitations for detection of antibodies against CSF from samples collected at early time points after infections.

## Supplementary Information

Below is the link to the electronic supplementary material.

Supplementary Material 1 (DOCX 16.2 KB)

Supplementary Material 2 (DOCX 16.4 KB)

## Data Availability

All data generated or analyzed during this study are included in this published article and its supplementary information files.
