# Supplementary material for: Blood swabs represent an alternative sample matrix for detection of antibodies against classical swine fever virus during surveillance in wild boar
Source: Vet Res Commun. 2026 May 25;50(4):346. doi: 10.1007/s11259-026-11292-3 (PMC13201338; doi:10.1007/s11259-026-11292-3)
Supplement: Supplementary file 1 — Supplementary Material 1 (DOCX 16.2 KB) [file 11259_2026_11292_MOESM1_ESM.docx]

**Titel:** Blood swabs represent an alternative sample matrix for detection of antibodies against Classical swine fever virus during surveillance in wild boar

**Journal:** Veterinary Research Communication

**Authors**: Denise Meyer, Sandra Blome, Lia Ebner and Paul Becher

**Corresponding authors:** Denise Meyer (denise.meyer@tiho-hannover.de) and Paul Becher (paul.becher@tiho-hannover.de); EU and WOAH Reference Laboratory for Classical Swine Fever, Institute of Virology, University of Veterinary Medicine Hannover, Buenteweg 17, 30559 Hannover, Germany

**Online Resource 1:** Characteristics of the EDTA and corresponding serum samples

| **Animal-ID** | **Virus inoculum** | **Genotype** | **Days post infection** |
| --- | --- | --- | --- |
| **397** | CSF1058 | 1.4 | 17 |
| **398** | CSF1058 | 1.4 | *0, 7, 14, 21, 28, 35 |
| **399** | CSF1058 | 1.4 | 35 |
| **429** | CSF1059 | 2.2 | *0, 7, 21, 28, 38 |
| **431** | CSF1059 | 2.2 | 21 |
| **434** | CSF1076 | 3.4 | *0, 7, 14, 21, 28, 35 |
| **435** | CSF1076 | 3.4 | *0, 7, 14, 21, 27 |
| **436** | CSF1076 | 3.4 | 21 |
| **437** | CSF1076 | 3.4 | 35 |
| **441** | CSF1059 | 2.2 | 21 |
| **443** | CSF0410 | 3.1 | 26 |
| **444** | CSF0410 | 3.1 | 34 |
| **445** | CSF0410 | 3.1 | 34 |
| **446** | CSF0410 | 3.1 | 34 |
| **447** | CSF0410 | 3.1 | 26 |

* = Serial-derived samples were included in the study. As part of the analysis of these serially obtained samples, day 27 or 28 after infection was examined as the last point in time, as the antibody level against CSFV was already high on that day.
