# Supplementary material for: Blood swabs represent an alternative sample matrix for detection of antibodies against classical swine fever virus during surveillance in wild boar
Source: Vet Res Commun. 2026 May 25;50(4):346. doi: 10.1007/s11259-026-11292-3 (PMC13201338; doi:10.1007/s11259-026-11292-3)
Supplement: Supplementary file 2 — Supplementary Material 2 (DOCX 16.4 KB) [file 11259_2026_11292_MOESM2_ESM.docx]

**Titel:** Blood swabs represent an alternative sample matrix for detection of antibodies against Classical swine fever virus during surveillance in wild boar

**Journal:** Veterinary Research Communication

**Authors**: Denise Meyer, Sandra Blome, Lia Ebner and Paul Becher

**Corresponding authors:** Denise Meyer (denise.meyer@tiho-hannover.de) and Paul Becher (paul.becher@tiho-hannover.de); EU and WOAH Reference Laboratory for Classical Swine Fever, Institute of Virology, University of Veterinary Medicine Hannover, Buenteweg 17, 30559 Hannover, Germany

**Online Resource 2:** Number of serum and blood swab samples tested positive, doubtful and negative during early seroconversion. Samples obtained at 7 (*n* = 4), 14 (*n* = 3), and 21 (*n* = 4) days post infection (dpi) were analyzed by two commercial CSF antibody ELISAs.

| Sample matrix and days post infection (dpi) | IDEXX CSF Ab ELISA | | | ID Screen CSF E2 Competition | | |
| --- | --- | --- | --- | --- | --- | --- |
|  | positive | doubtful | negative | positive | doubtful | negative |
| Serum, 7 dpi | 0 | 0 | 4 | 0 | 0 | 4 |
| Blood swab, 7 dpi | 0 | 0 | 4 | 0 | 0 | 4 |
| Serum, 14 dpi | 2 | 1 | 0 | 2 | 1 | 0 |
| Blood swab, 14 dpi | 2 | 0 | 1 | 0 | 0 | 3 |
| Serum, 21 dpi | 4 | 0 | 0 | 4 | 0 | 0 |
| Blood swab, 21 dpi | 4 | 0 | 0 | 4 | 0 | 0 |
